# Supplementary material for: Resolving the full spectrum of human genome variation using Linked-Reads
Source: Genome Res. 2019 Apr;29(4):635–45. doi: 10.1101/gr.234443.118 (PMC6442396; doi:10.1101/gr.234443.118)
Supplement: Supplemental Material [file supp_gr.234443.118_Supplemental_longranger-2.2.zip › longranger-2.2.tar.bz2!/lr2.2-public/lib/stan/cmdstan/stan/doc/api/html/classstan_1_1json_1_1json__data__handler.html]

Stan: stan::json::json\_data\_handler Class Reference


|  |  |
| --- | --- |
|  | Stan  2.5.0  probability, sampling & optimization |


- Main Page
- Related Pages
- Namespaces
- Classes
- Files

- Class List
- Class Index
- Class Hierarchy
- Class Members

All Classes Namespaces Files Functions Variables Typedefs Enumerations Enumerator Friends Macros Pages

- stan
- json
- json\_data\_handler

Public Member Functions |
List of all members

stan::json::json\_data\_handler Class Reference

A `json_data_handler` is an implementation of a `json_handler` that restricts the allowed JSON text a set of Stan variable declarations in JSON format.
More...

`#include <json_data_handler.hpp>`

Inheritance diagram for stan::json::json\_data\_handler:

|  |  |
| --- | --- |
| Public Member Functions | |
|  | json\_data\_handler (vars\_map\_r &vars\_r, vars\_map\_i &vars\_i) |
|  | Construct a json\_data\_handler object. More... |
|  | |
| void | start\_text () |
|  | Handle the the start of the text. More... |
|  | |
| void | end\_text () |
|  | Handle the the end of the text. More... |
|  | |
| void | start\_array () |
|  | Handle the start of an array. More... |
|  | |
| void | end\_array () |
|  | Handle the end of an array. More... |
|  | |
| void | start\_object () |
|  | Handle the start of an object. More... |
|  | |
| void | end\_object () |
|  | Handle the end of an object. More... |
|  | |
| void | null () |
|  | Handle the null literal value. More... |
|  | |
| void | boolean (bool p) |
|  | Handle the boolean literal value of the specified polarity. More... |
|  | |
| void | string (const std::string &s) |
|  | Handle the specified string value. More... |
|  | |
| void | key (const std::string &key) |
|  | Handle the specified object key. More... |
|  | |
| void | number\_double (double x) |
|  | Handle a the specified double-precision floating point value. More... |
|  | |
| void | number\_long (long n) |
|  | Handle a the specified long integer value. More... |
|  | |
| void | number\_unsigned\_long (unsigned long n) |
|  | Handle a the specified unsigned long integer value. More... |
|  | |
| void | save\_current\_key\_value\_pair () |
|  | |
| void | incr\_dim\_size () |
|  | |
| template<typename T > | |
| void | to\_column\_major (std::vector< T > &cm\_vals, const std::vector< T > &rm\_vals, const std::vector< size\_t > &dims) |
|  | |
| void | set\_last\_dim () |
|  | |
| size\_t | convert\_offset\_rtl\_2\_ltr (size\_t rtl\_offset, const std::vector< size\_t > &dims) |
|  | |
| Public Member Functions inherited from stan::json::json\_handler | |
|  | json\_handler () |
|  | |
|  | ~json\_handler () |
|  | |

## Detailed Description

A `json_data_handler` is an implementation of a `json_handler` that restricts the allowed JSON text a set of Stan variable declarations in JSON format.

Each Stan variable consists of a JSON key : value pair. The key is a string and the value is either a single numeric scalar value or a JSON array of numeric values.

The `json_data_handler` checks that the top-level JSON object contains a set of name-value pairs where the values can be either numeric scalar objects or or numeric arrays of any dimensionality. Arrays must be rectangular. Empty arrays are not allowed, nor are arrays of empty arrays. The strings "inf" and "-inf" are mapped to positive and negative infinity, respectively.

Definition at line 54 of file json\_data\_handler.hpp.

## Constructor & Destructor Documentation

|  |  |  |  |
| --- | --- | --- | --- |
| stan::json::json\_data\_handler::json\_data\_handler | ( | vars\_map\_r & | *vars\_r*, |
|  |  | vars\_map\_i & | *vars\_i* |
|  | ) |  |  |

inline

Construct a json\_data\_handler object.

**Warning:** This method does not close the input stream.

Parameters
:   |  |  |
    | --- | --- |
    | vars\_r | - name-value map for real-valued variables |
    | vars\_i | - name-value map for int-valued variables |

Definition at line 102 of file json\_data\_handler.hpp.

## Member Function Documentation

|  |  |  |  |  |  |
| --- | --- | --- | --- | --- | --- |
| void stan::json::json\_data\_handler::boolean | ( | bool | *p* | ) |  |

inlinevirtual

Handle the boolean literal value of the specified polarity.

Parameters
:   |  |  |
    | --- | --- |
    | p | polarity of boolean |

Reimplemented from stan::json::json\_handler.

Definition at line 177 of file json\_data\_handler.hpp.

|  |  |  |  |
| --- | --- | --- | --- |
| size\_t stan::json::json\_data\_handler::convert\_offset\_rtl\_2\_ltr | ( | size\_t | *rtl\_offset*, |
|  |  | const std::vector< size\_t > & | *dims* |
|  | ) |  |  |

inline

Definition at line 306 of file json\_data\_handler.hpp.

|  |  |  |  |  |
| --- | --- | --- | --- | --- |
| void stan::json::json\_data\_handler::end\_array | ( |  | ) |  |

inlinevirtual

Handle the end of an array.

Reimplemented from stan::json::json\_handler.

Definition at line 139 of file json\_data\_handler.hpp.

|  |  |  |  |  |
| --- | --- | --- | --- | --- |
| void stan::json::json\_data\_handler::end\_object | ( |  | ) |  |

inlinevirtual

Handle the end of an object.

Reimplemented from stan::json::json\_handler.

Definition at line 166 of file json\_data\_handler.hpp.

|  |  |  |  |  |
| --- | --- | --- | --- | --- |
| void stan::json::json\_data\_handler::end\_text | ( |  | ) |  |

inlinevirtual

Handle the the end of the text.

Reimplemented from stan::json::json\_handler.

Definition at line 115 of file json\_data\_handler.hpp.

|  |  |  |  |  |
| --- | --- | --- | --- | --- |
| void stan::json::json\_data\_handler::incr\_dim\_size | ( |  | ) |  |

inline

Definition at line 279 of file json\_data\_handler.hpp.

|  |  |  |  |  |  |
| --- | --- | --- | --- | --- | --- |
| void stan::json::json\_data\_handler::key | ( | const std::string & | *s* | ) |  |

inlinevirtual

Handle the specified object key.

Parameters
:   |  |  |
    | --- | --- |
    | s | String object key to handle. |

Reimplemented from stan::json::json\_handler.

Definition at line 203 of file json\_data\_handler.hpp.

|  |  |  |  |  |
| --- | --- | --- | --- | --- |
| void stan::json::json\_data\_handler::null | ( |  | ) |  |

inlinevirtual

Handle the null literal value.

Reimplemented from stan::json::json\_handler.

Definition at line 171 of file json\_data\_handler.hpp.

|  |  |  |  |  |  |
| --- | --- | --- | --- | --- | --- |
| void stan::json::json\_data\_handler::number\_double | ( | double | *x* | ) |  |

inlinevirtual

Handle a the specified double-precision floating point value.

Parameters
:   |  |  |
    | --- | --- |
    | x | Value to handle. |

Reimplemented from stan::json::json\_handler.

Definition at line 209 of file json\_data\_handler.hpp.

|  |  |  |  |  |  |
| --- | --- | --- | --- | --- | --- |
| void stan::json::json\_data\_handler::number\_long | ( | long | *n* | ) |  |

inlinevirtual

Handle a the specified long integer value.

Parameters
:   |  |  |
    | --- | --- |
    | n | Value to handle. |

Reimplemented from stan::json::json\_handler.

Definition at line 221 of file json\_data\_handler.hpp.

|  |  |  |  |  |  |
| --- | --- | --- | --- | --- | --- |
| void stan::json::json\_data\_handler::number\_unsigned\_long | ( | unsigned long | *n* | ) |  |

inlinevirtual

Handle a the specified unsigned long integer value.

Parameters
:   |  |  |
    | --- | --- |
    | n | Value to handle. |

Reimplemented from stan::json::json\_handler.

Definition at line 231 of file json\_data\_handler.hpp.

|  |  |  |  |  |
| --- | --- | --- | --- | --- |
| void stan::json::json\_data\_handler::save\_current\_key\_value\_pair | ( |  | ) |  |

inline

Definition at line 241 of file json\_data\_handler.hpp.

|  |  |  |  |  |
| --- | --- | --- | --- | --- |
| void stan::json::json\_data\_handler::set\_last\_dim | ( |  | ) |  |

inline

Definition at line 296 of file json\_data\_handler.hpp.

|  |  |  |  |  |
| --- | --- | --- | --- | --- |
| void stan::json::json\_data\_handler::start\_array | ( |  | ) |  |

inlinevirtual

Handle the start of an array.

Reimplemented from stan::json::json\_handler.

Definition at line 119 of file json\_data\_handler.hpp.

|  |  |  |  |  |
| --- | --- | --- | --- | --- |
| void stan::json::json\_data\_handler::start\_object | ( |  | ) |  |

inlinevirtual

Handle the start of an object.

Reimplemented from stan::json::json\_handler.

Definition at line 158 of file json\_data\_handler.hpp.

|  |  |  |  |  |
| --- | --- | --- | --- | --- |
| void stan::json::json\_data\_handler::start\_text | ( |  | ) |  |

inlinevirtual

Handle the the start of the text.

Reimplemented from stan::json::json\_handler.

Definition at line 109 of file json\_data\_handler.hpp.

|  |  |  |  |  |  |
| --- | --- | --- | --- | --- | --- |
| void stan::json::json\_data\_handler::string | ( | const std::string & | *s* | ) |  |

inlinevirtual

Handle the specified string value.

Parameters
:   |  |  |
    | --- | --- |
    | s | String value to handle. |

Reimplemented from stan::json::json\_handler.

Definition at line 183 of file json\_data\_handler.hpp.

template<typename T >

|  |  |  |  |
| --- | --- | --- | --- |
| void stan::json::json\_data\_handler::to\_column\_major | ( | std::vector< T > & | *cm\_vals*, |
|  |  | const std::vector< T > & | *rm\_vals*, |
|  |  | const std::vector< size\_t > & | *dims* |
|  | ) |  |  |

inline

Definition at line 289 of file json\_data\_handler.hpp.

---

The documentation for this class was generated from the following file:

- src/stan/io/json/json\_data\_handler.hpp


---

[ Stan Home Page ]

*© 2011–2014,
Stan Development Team.*
